# Prevalence of HIV drug-resistance mutations in HIV-infected Mexican patients heavily experienced to antiretroviral therapy

**DOI:** 10.1186/1758-2652-13-S4-P139

**Published:** 2010-11-08

**Authors:** JE Gaytán-Martínez, JA Mata-Marín, F Gutierrez-Escolano, L Pérez-Saleme, S Treviño-Pérez, JG Vázquez-Rosales, N Matías-Juan, RD Díaz-Ramos, R Arias-Flores

**Affiliations:** 1 Hospital de Infectología, Infectious diseases, Mexico City, Mexico; 2 HGZ-72/IMSS, Internal Medicine, Mexico City, Mexico; 3 Hospital de Especialidades "Siglo XXI" National Medical Center, Infectious diseases, Mexico City, Mexico; 4 Hospital Carlos McGregor/IMSS, Internal Medicine, Mexico City, Mexico; 5 Hospital de pediatria "Siglo XXI" National Medical Center, Pediatric Infectious Diseases, Mexico City, Mexico; 6 Hospital de infectología "La Raza" National Medical Center, Pediatric Infectious Diseases, Mexico City, Mexico; 7 IMSS, Infectious diseases, Mexico City, Mexico; 8 Hospital de infectología "La Raza" National Medical Center, Clinical Epidemiology, Mexico City, Mexico

## Background

A significant number of HIV Mexican infected people are in virological failure, many of them are multiply experienced to antiretroviral therapy.

## Objective

To assess the prevalence of resistance mutations in HIV Mexican infected patients heavily experienced to antiretroviral therapy (ART).

## Materials and methods

A transversal observational study of resistance profiles in strains from HIV-1 infected patients multiple experienced to ART was analyzed by a national committee of HIV drugs resistance in Mexico (GERA-1 IMSS). Mutations were defined according Stanford Resistance Database.

## Results

We assessed 178 subjects, mean age (SD ±) of our subjects was 41.9 ± 11.9; 91% where male, who failed 2 to 15 ART regimen (median 5). CDC status was A 20%, B 21% and C 59%; 4% were coinfected with HBV and 2% were infected with HCV. Month median drug exposure was 121 (range 12-219). Ninety nine (55.6%) had triple class drug mutations, 170 (95.5%) had NRTI mutations, 113 (63.5%) had NNRTI mutations and 173 (97.2%) had PIs mutations. The most frequent NRTI mutation belonged to TAM pathways, 74 (41.6%) to TAM1 and 40 (22.5%) to TAM2. These mutations were 215Y/F 139 (77.7%), M41L 111 (62%), D67N 97 (54.2%), L210W 85 (47.5%), K219E/Q 60 (33.5%) and K70R 53 (29.6%) and reflect the extensive use of ZDV and d4T. K65R was found in 8 (4.4%), Q151M was found in 2 (1.1%) and insertion 69 in 1 (.5 %). The most frequent NNRTI mutation was K103N in 57 (31.8%) followed by Y181C 40 (22.3%), G190A/S 40 (22.3%), V108I 18 (10%), K101E 16 (8.9%), L100I 13 (7.3%), Y188L 11(6.1%), A98G 10(5.6%), V90I 9 (5%); 14 (8%) of them had resistance to ETV. Most common PI major mutation were L10V/F 144 (80%), I54L/M/V 126 (70.4%), M36I/L 102 (57%), L90M 101 (56%), 82A/T 92 (51.4%), M46I/L 91 (50.8%), L33F/I 69 (38.5%), I84V 64 (35%) and others. 15 (8.4%) of them had resistance to DRV and 33 (18.5%) to TPV.

## Conclusions

Long term exposure to ART and fluctuating adherence has led to the selection of multiresistant strains in Mexican HIV infected patients, many of them have resistance mutation for ART of new generation.

